# The impact of Epstein‐Barr virus latent membrane protein 2A on the production of B cell activating factor of the tumor necrosis factor family (BAFF), APRIL and their receptors

**DOI:** 10.1002/iid3.729

**Published:** 2022-10-26

**Authors:** Kevin Madayag, Ryan Incrocci, Michelle Swanson‐Mungerson

**Affiliations:** ^1^ Department of Biomedical Sciences College of Graduate Studies Downers Grove Illinois USA; ^2^ Department of Microbiology and Immunology, College of Graduate Studies Midwestern University Downers Grove Illinois USA

**Keywords:** APRIL, BAFF, EBV, LMP2A, lymphoma, TNF family ligands

## Abstract

**Introduction:**

Epstein‐Barr virus (EBV) establishes a lifelong infection in human B cells where the virus consistently expresses Latent Membrane Protein 2A (LMP2A) to promote B cell survival. A prior study indicates that LMP2A may increase the production of the pro‐survival factor, B cell Activating Factor of the tumor necrosis factor family (BAFF), which could also indirectly increase B cell survival. The current study sought to extend these findings and determine if LMP2A increased BAFF production and/or the responsiveness of LMP2A‐expressing cells to this cytokine.

**Methods:**

Four independently derived LMP2A‐negative and ‐positive B cell lymphoma cell lines were analyzed for BAFF and APRIL levels by both ELISA and Western Blot analysis. Additionally, flow cytometric analysis measured any LMP2A‐dependent changes in the receptors for BAFF and APRIL (BAFF‐R, transmembrane activator and calcium‐modulator and cyclophilin ligand interactor [TACI], B cell maturation antigen [BCMA]) in both LMP2A‐negative and ‐positive B cell lymphoma cell lines.

**Results:**

In contrast to previous reports, our data indicate that LMP2A does not increase the expression of BAFF or APRIL by Western blot analysis or ELISA. Additionally, flow cytometric analysis indicates that LMP2A does not influence the expression of the receptors for BAFF and APRIL: TACI, BAFF‐R, and BCMA.

**Conclusion:**

Therefore, these data suggest that while EBV utilizes other latency proteins to regulate BAFF production, EBV does not appear to use LMP2A to enhance BAFF‐or APRIL‐dependent survival to promote EBV latency.

## Graphical abstract

In contrast to previous reports, Latent Membrane Protein 2A (LMP2A) does not increase B cell Activating Factor of the tumor necrosis factor family (BAFF) or APRIL production and/or its receptors. Since LMP2A is expressed in Epstein‐Barr virus‐associated autoimmune diseases, this suggests that the BAFF/APRIL axis is not influenced by LMP2A to promote autoimmune disease.

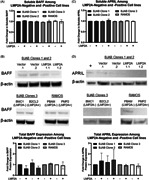

## 1 INTRODUCTION

Epstein‐Barr virus (EBV) is a successful gamma herpesvirus that infects over 90% of the population worldwide.^1^, ^2^ For the majority of the population, there is little consequence of EBV infection, while others will manifest with infectious mononucleosis^3^ or EBV‐associated malignancies, including Burkitt's lymphoma, Hodgkin's lymphoma, and posttransplant lymphoproliferative disease.^4^ Furthermore, EBV is associated with the development of numerous autoimmune diseases, such as arthritis, systemic lupus erythematosus, and multiple sclerosis.^5^ Therefore, understanding how EBV contributes to the survival and dysregulation of normal B cell function could significantly change our approach to treating EBV‐associated autoimmune diseases and malignancies.

Multiple EBV latency genes are implicated in dysregulating normal B cell function and promoting tumor development. Of note, the EBV latency genes, latent membrane protein 1 (LMP1) is directly oncogenic^6^, ^7^ and latent membrane protein 2A (LMP2A) enhances B cell proliferation,^8^ survival,^9^, ^10^ antibody production,^8^ tumor development,^11^, ^12^ and bypasses normal checkpoints that inactivate self‐reactive B cells.^13^ LMP1 performs these functions through the activation of PI3K and NF‐kB,^6^ while LMP2A promotes these functions by mimicking the B cell receptor through its ITAM motifs to activate SYK, RAS, and PI3K.^10^, ^14^, ^15^

During normal latency, LMP2A promotes B cell survival^10^, ^14^ by directly increasing pro‐survival transcription factors such as NF‐kB, BCL‐2, and BCL‐xL^10^, ^12^, ^16^ and indirectly increasing pro‐survival cytokines, such as IL‐10.^9^, ^17^ It was also demonstrated that LMP2A may increase the production of the pro‐survival cytokine B cell activating factor of the tumor necrosis factor family (BAFF),^18^ which is not overly surprising since BAFF induction is regulated by NF‐kB and IL‐10.^19^, ^20^ The ability of LMP2A to increase this cytokine and/or the responsiveness of B cells to this cytokine may significantly impact the survival of latently EBV‐infected cells.

BAFF is a cytokine that is produced by stromal cells, myeloid cells and dendritic cells.^21^ BAFF promotes the maturation of immature B cells into naïve mature B cells^22^, ^23^ and the survival of anergic autoreactive B cells.^24^ However, BAFF can be aberrantly expressed by virally‐infected B cells and B cell lymphomas^18^, ^19^, ^25^ that could promote pathology. BAFF binds three receptors, which are B cell maturation antigen (BCMA), transmembrane activator and calcium‐modulator and cyclophilin ligand interactor (TACI) and BAFF receptor (BAFF‐R).^22^, ^25^, ^26^ All three receptors are found on B cells, with BAFF‐R being exclusively expressed on B cells.^26^, ^27^ Therefore, factors that influence BAFF levels and/or receptors for BAFF could have a significant impact on B cell associated autoimmune diseases and B cell tumor formation.

Due to BAFF's importance in both tumorigenesis and autoimmunity, there is interest whether EBV latency proteins enhance BAFF production by B cells. A previous study indicates that the EBV protein LMP1 increases BAFF production and suggested that LMP2A may increase BAFF production, albeit to a lesser extent than LMP1.^18^ Therefore, we sought to confirm whether LMP2A increases the production of BAFF and/or the responsiveness of B cells to BAFF or the related cytokine APRIL. Even though LMP2A increases NF‐kB activation,^16^, ^28^ which can induce the aberrant production of BAFF in B cells,^18^, ^19^ our data indicate that LMP2A does not increase BAFF or APRIL levels. Furthermore, even though LMP2A is a BCR mimic^10^, ^15^ and BCR signaling induces the expression of receptors for BAFF,^29^ LMP2A does not regulate the level of receptors that bind BAFF and/or APRIL. Taken together, the data suggest that EBV does not appear to use LMP2A to regulate BAFF production or responsiveness to increase the survival of latently‐infected B cells.

## 2 MATERIALS AND METHODS

### 2.1 Cell lines

The BJAB and RAMOS cell lines were generously provided by Dr. Richard Longnecker (Northwestern University) and have been described previously by our laboratory and others.^9^, ^30^ All cell lines were maintained in appropriate selective media for all experiments and LMP2A expression was confirmed for all cell lines (Supporting Information: Figure 1).

### 2.2 Flow cytometry

Flow cytometry was performed as described previously.^31^ Briefly, LMP2A‐negative or ‐positive B cell lines (0.5 × 10^6^ cells) were stained with the following antibodies (0.2 ug/ml): APC‐conjugated mouse anti‐human BAFF‐R (Biolegend, San Diego, CA #316916), APC‐conjugated mouse anti‐human BCMA (Biolegend, San Diego, CA #357505), APC‐conjugated rat anti‐human TACI (BD Pharmingen, Franklin Lakes, NJ #562345), or the appropriate isotype control. After 30 min, all cells were washed three times in PBS and analyzed on a Becton Dickinson FACSCalibur and Cellquest software (BD Biosciences).

### 2.3 Western blot analysis

LMP2A‐negative or ‐positive BJAB and RAMOS cell lines (2 × 10^6^ cells) were analyzed by Western Blot analysis as described previously.^9^, ^32^ The K562 cell lysate (ProSci Inc., Poway, CA #1204) was used as a positive control for the presence of APRIL protein. Pre‐determined dilutions of polyclonal anti‐BAFF (EMD Millipore, Burlington, MA #AB16530) or anti‐APRIL (ProSci Inc., Poway, CA #2223) antibodies were used followed by a dilution of polyclonal rabbit anti‐β actin antibody (Thermo Fisher Scientific, Rockford, IL #PA1‐16889) in blocking buffer containing 5% dry milk. After washing, goat antirabbit IgG HRP‐conjugated antibody (Thermo Fisher Scientific, Rockford, IL #31462) in blocking buffer containing 5% dry milk was added, followed by washing in TBST and visualized using chemiluminescence (GE Healthcare Biosciences, Marlborough, MA #RPN2235). All blots were imaged using a Bio‐Rad Chemi Doc MP Imaging system (Bio‐Rad, Hercules, CA #1708280) and BioRad Image Lab 5.2.1 Imaging Software (Bio‐Rad, Hercules, CA #1709690). Image analysis and quantification was performed using ImageJ (NIH).

### 2.4 ELISA

LMP2A‐negative and ‐positive B cell lines (5 × 10^4^) were incubated in a 96 well plate for 24 h at 37°C/5% CO_2_. Supernatants were isolated and analyzed for soluble BAFF using a BAFF ELISA kit (R and D Systems, Minneapolis, MN #DBLYS0B) or an APRIL ELISA kit (Biolegend, San Diego, CA #439307) according to manufacturers' instructions. ELISA plates were read on a Thermo Multiskan FC spectrophotometer (Thermo Fisher, Waltham, MA #51119000) at an absorbance of 450 nm.

### 2.5 Statistics

All experiments were performed 2–3 times. When at least three experiments were performed, the data from each independent experiment was combined and analyzed by one‐way analysis of variance to determine if there was statistical significance within the experiment. All analysis was performed using Graphpad Prism Software (version 9.0).

## 3 RESULTS

**Figure 1:**
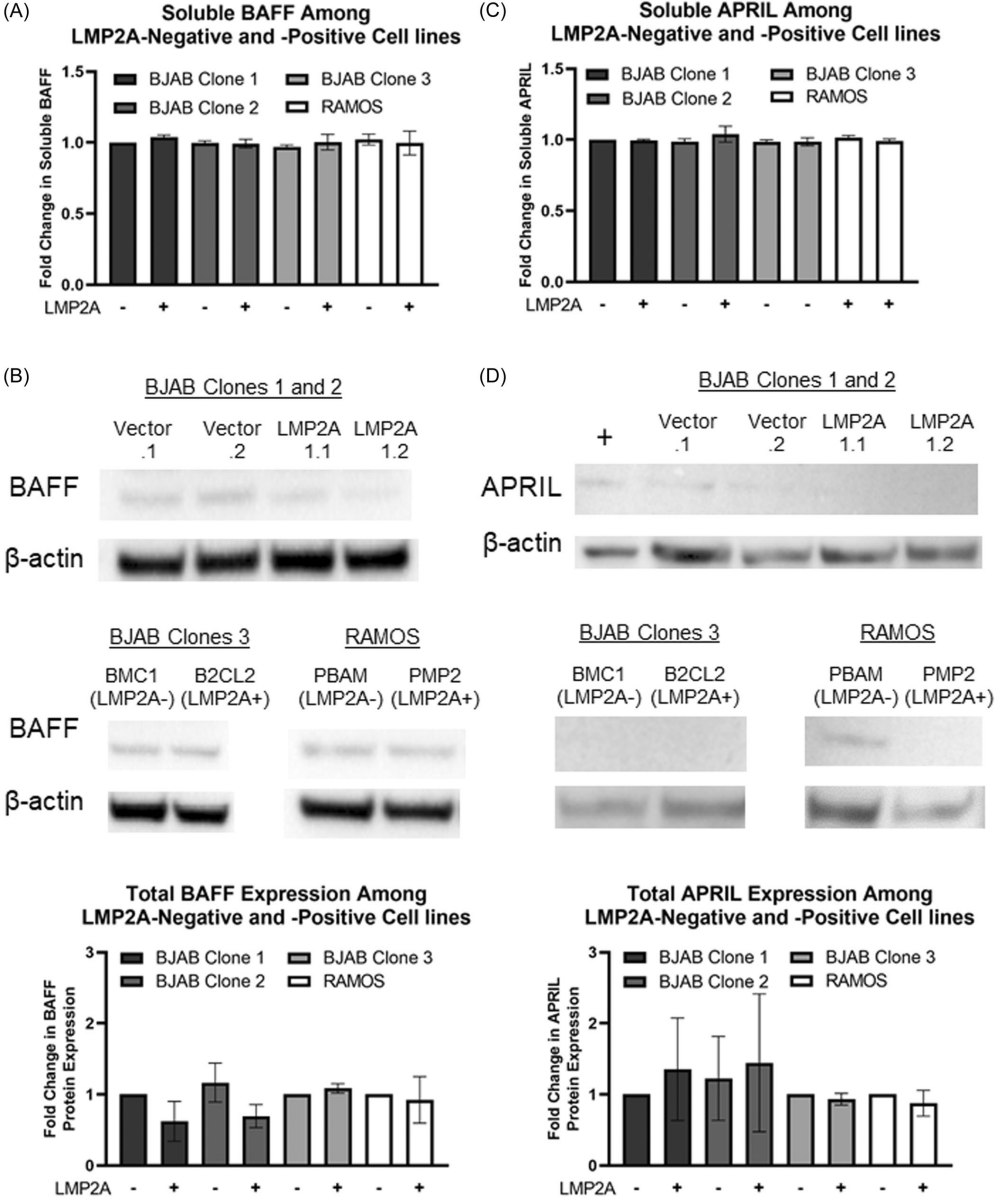
LMP2A does not affect the production BAFF or APRIL levels in B cell lymphoma cells. LMP2A‐negative (BJAB Vector.1, BJAB Vector.2, BJAB BCM1, RAMOS‐PBAM) and LMP2A‐positive (BJAB LMP2A1.1, BJAB LMP2A1.2, BJAB B2CL2, RAMOS‐PMP2) B cell lines were incubated for 48 h before supernatants were isolated and analyzed by ELISA for soluble BAFF (A) or APRIL (C) protein lysates were isolated from LMP2A‐negative and ‐positive B cell lines and Western Blot was used to determine total BAFF (B) or APRIL (D) Experiments are either a combination of three experiments for (A, C) or representative of two experiments for (B, D) and a bar graph demonstrating the results of combining the representative Western blots. All data was analyzed statistically as described in the Section 2 and was found to not reach a *p* < .05. BAFF, B cell Activating Factor of the tumor necrosis factor family; LMP2A, Latent Membrane Protein 2A.

Based on a previous report suggesting that LMP2A increases BAFF production in RAMOS cells,^18^ we sought to confirm and extend the previous group's findings. We tested BAFF levels by ELISA in the supernatants from LMP2A‐negative and ‐positive RAMOS cell lines, as well as three unique clones of LMP2A‐negative and ‐positive BJAB cell lines. In contrast to previous findings, LMP2A did not increase soluble BAFF production in either RAMOS cell lines or any of the BJAB cell lines tested (Figure 1A). Since BAFF can also be retained in a membrane‐bound form, we isolated total protein from RAMOS and BJAB cell lines and determined BAFF levels by Western Blot analysis. As shown in Figure 1B, LMP2A did not increase total levels of BAFF protein.

Since APRIL binds to similar receptors as BAFF, we determined if LMP2A increases APRIL production. When LMP2A‐negative and ‐positive cell lines were analyzed for APRIL production, LMP2A did not increase APRIL production by ELISA (Figure 1C) and was negligibly detectably in all cell lines by Western blot analysis, even though our positive control lysates from the K562 cell line were positive for APRIL (Figure 1D). Our Western blot data are not overly surprising, since nonactivated B cells do not produce considerable amounts of APRIL^33^ and we detected very low levels of APRIL by the more sensitive ELISA assay.

**Figure 2:**
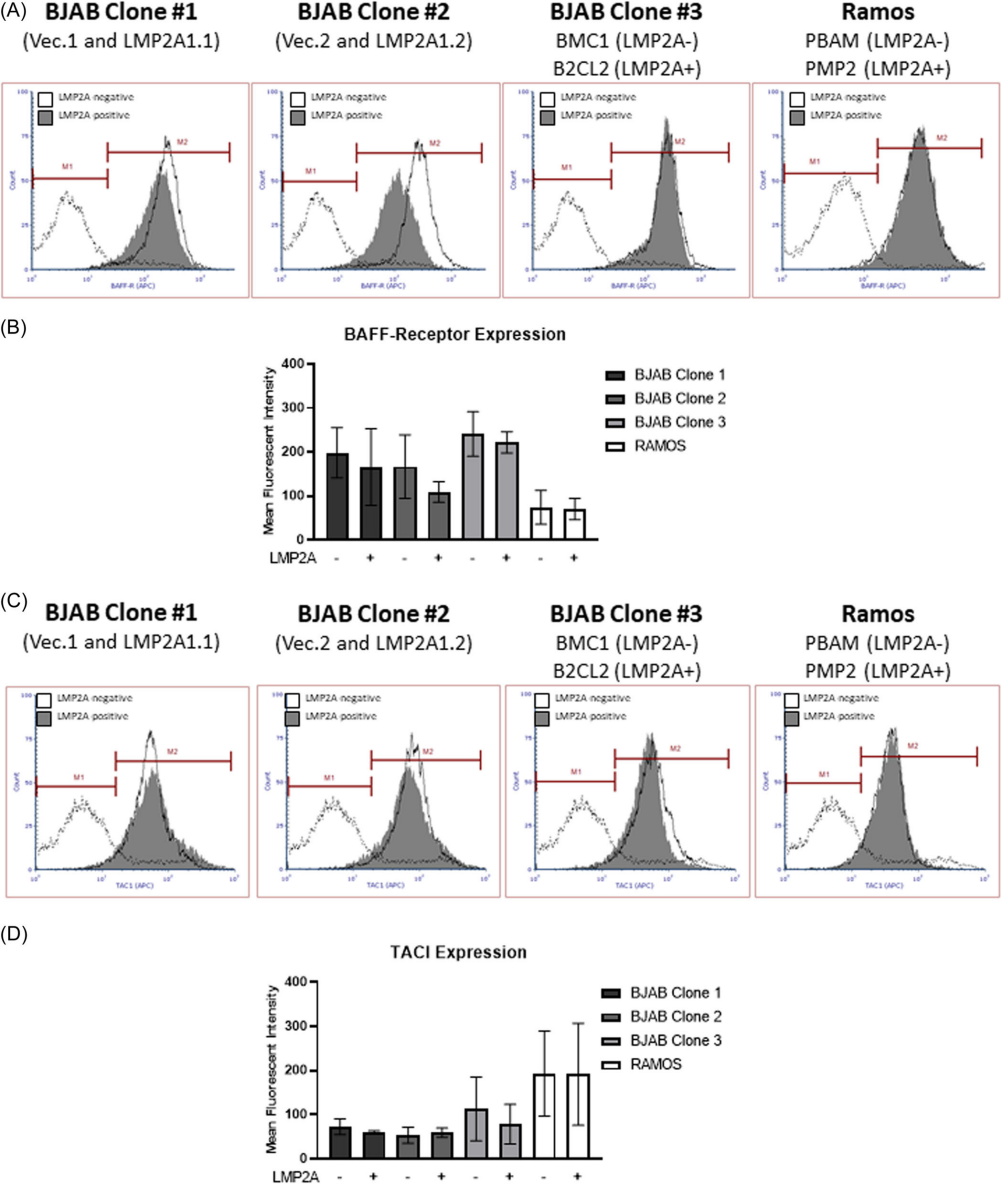
LMP2A does not affect the level of BAFF‐R or TACI. LMP2A‐negative (BJAB Vector.1, BJAB Vector.2, BJAB BCM1, RAMOS‐PBAM) and LMP2A‐positive (BJAB LMP2A1.1, BJAB LMP2A1.2, BJAB B2CL2, RAMOS‐PMP2) B cell lines were analyzed for flow cytometry for BAFF‐receptor (A, B) or TACI (C, D) the data in (A, C) are representative flow cytometry plots with the shaded histograms representative of LMP2A‐positive cells and nonshaded histograms representative of LMP2A‐negative cells. The histogram representative with the dotted line represents the staining pattern of the isotype control for those cell lines. (B, D) represent combinations of the mean fluorescence intensity (MFI) from three experiments analyzing BAFF‐R or TACI, respectively with a *p* ≥.05. BCMA was not detected on any of the cell lines analyzed (data not shown). BAFF, B cell Activating Factor of the tumor necrosis factor family; BCMA, B cell maturation antigen; LMP2A, Latent Membrane Protein 2A; TACI, transmembrane activator and calcium‐modulator and cyclophilin ligand interactor.

We subsequently tested the possibility that LMP2A increases the responsiveness of B cells to these cytokines by increasing the levels of receptor for BAFF and APRIL. Previous studies show that signaling through the BCR increases BAFF‐R expression^29^ and since LMP2A mimics BCR signaling,^10^, ^15^ we hypothesized that LMP2A may increase BAFF‐R expression. However, LMP2A did not significantly change BAFF‐R expression in any of the B cell lines tested by flow cytometry (Figure 2A), which was confirmed when multiple experiments were combined (Figure 2B). TACI is another receptor that binds to BAFF and APRIL and is found on multiple B cell subsets.^34^, ^35^, ^36^, ^37^ Once again, LMP2A did not influence TACI levels (Figure 2C,D). Finally, the third receptor for BAFF and APRIL is BCMA^34^ and has been found on Hodgkin's lymphoma and non‐Hodgkin's lymphoma,^38^ which can be EBV‐associated.^4^ However, we were unable to detect BCMA receptor on any of the cell lines tested (data not shown). Taken together, our data indicate that LMP2A neither increases the production of BAFF and APRIL nor enhances the levels of the receptors for these cytokines.

## 4 DISCUSSION

Previous studies demonstrated that LMP1 from EBV enhanced both BAFF and APRIL levels in an NF‐kB‐dependent manner and suggested that LMP2A may enhance levels of BAFF in RAMOS B cell lines.^18^ Since BAFF is an important cytokine in promoting both cell survival and autoimmune diseases that are associated with EBV infections,^21^, ^39^, ^40^ we tested whether LMP2A enhanced BAFF production and/or BAFF responsiveness in B cells.

Our data indicate that LMP2A does not increase BAFF production in RAMOS B cells, or other B cell lymphoma lines (Figure 1). It is possible that the level of LMP2A expression was lower in our LMP2A‐expressing RAMOS cells in comparison to the other group's RAMOS cells, since they were generated by different mechanisms.^18^, ^30^ This could imply that a minimum threshold of LMP2A signaling is required to increase BAFF expression. However, we do not feel that this is likely, since we tested four different independently derived cell lines that have previously demonstrated LMP2A‐medited effects^9^, ^17^, ^28^ and express LMP2A levels similar to EBV‐generated lymphoblastoid cell lines (Incrocci et al.^9^ and Supporting Information: Figure 1). Additionally, since LMP2A increased IL‐10 production^9^ and IL‐10 increases BAFF production,^20^ we hypothesized that the LMP2A‐dependent increase may indirectly increase BAFF production. However, the data did not support this hypothesis. One potential reason for the different outcomes of the study is that the LMP2A‐dependent increase in IL‐10 levels in our system may not be sufficient to increase BAFF production. For example, a previous study treated PBMCs with 100 ng/ml of IL‐10 to increase both membrane bound and soluble BAFF.^20^ This amount is 1000‐fold higher than the amount of IL‐10 induced by LMP2A‐expressing cell lines.^9^ Thus, the difference in IL‐10 levels and the cell type analyzed may explain why our data contrast with a previous report in regard to the ability of LMP2A to increase BAFF levels.

Additionally, previous reports indicate that BCR signaling results in the upregulation in BAFF‐receptor expression.^29^ Since LMP2A mimics BCR signaling, we reasoned that LMP2A would also increase BAFF‐R levels. However, our data indicate that this is not the case. This may be due to the fact that LMP2A signaling is more similar to the tonic signal induced by the BCR in a resting B cell in comparison to the activating signal that increases BAFF‐R expression.^31^

Therefore, our data indicate that EBV does not appear to use LMP2A to increase the production of or the responsiveness to BAFF and/or the related cytokine, APRIL. Other latency genes, such as LMP1, do impact BAFF production,^18^ so it is possible that EBV uses this cytokine to promote survival of latently‐infected B cells. One limitation of our studies is that we analyzed the impact of LMP2A on these pro‐survival factors and receptors in the absence of other EBV viral proteins. While we think that it is unlikely, it is possible that LMP2A may synergize with other EBV proteins to enhance the production of BAFF and/or APRIL in the context of the entire virus. Therefore, future studies that analyze the expression of BAFF and/or APRIL in the context of virally infected cell may reveal this possibility. With the approval of Belimumab, as a therapeutic anti‐BAFF monoclonal antibody for treating autoimmune diseases, additional studies that analyze the interplay of BAFF and EBV could identify novel approaches to treating EBV‐associated diseases.

## Supporting information

Supplementary information.

## Data Availability

The data that support the findings of this study are available from the corresponding author upon reasonable request.
