# Supplementary figures and images for: The impact of Epstein‐Barr virus latent membrane protein 2A on the production of B cell activating factor of the tumor necrosis factor family (BAFF), APRIL and their receptors
Source: Immun Inflamm Dis. 2022 Oct 26;10(11):e729. doi: 10.1002/iid3.729 (PMC9597489; doi:10.1002/iid3.729)

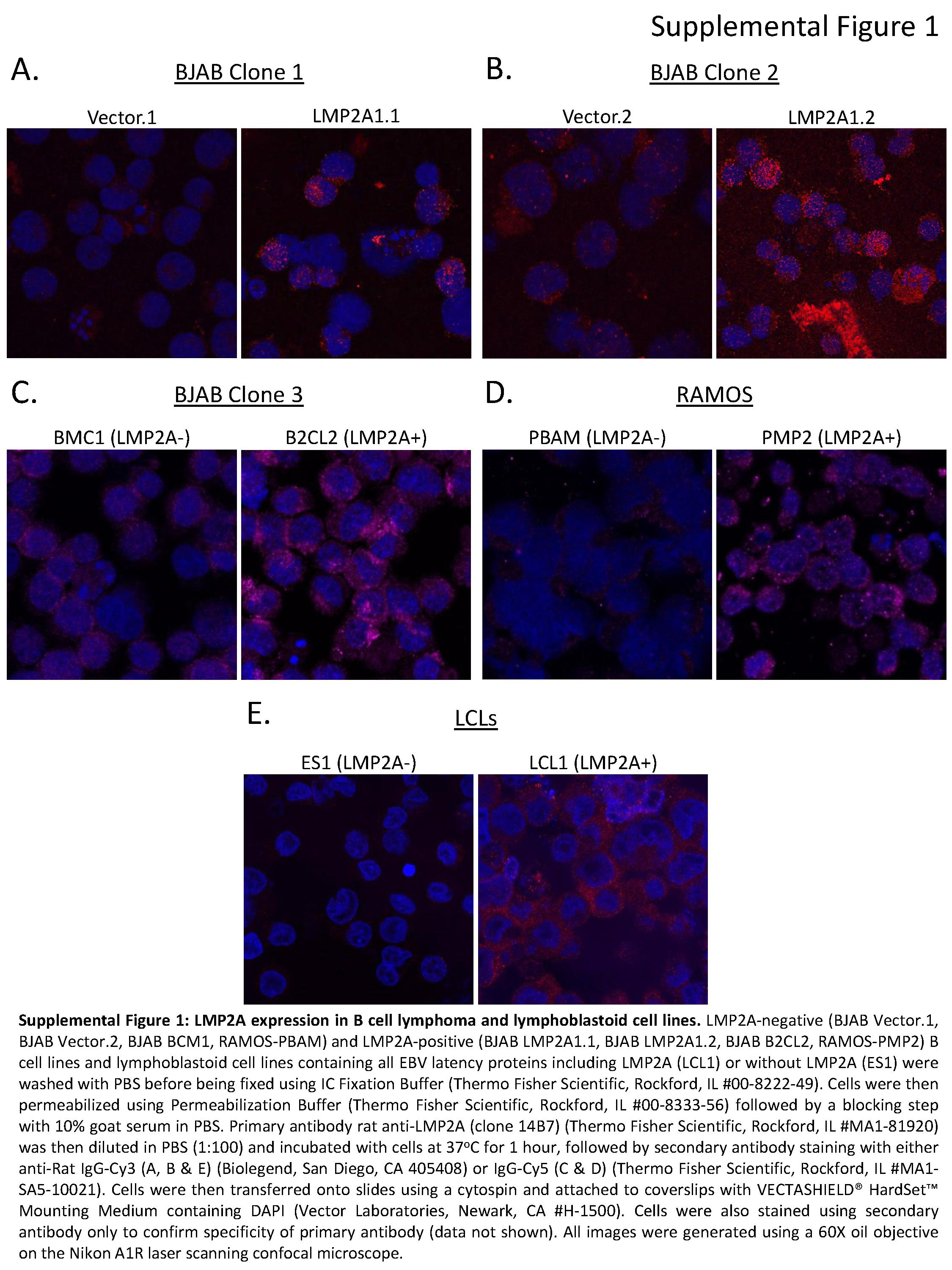

Supplement: Supplementary file 1 — Supplementary information. [file IID3-10-e729-s001.tif]
